# Suprabasin-null mice retain skin barrier function and show high contact hypersensitivity to nickel upon oral nickel loading

**DOI:** 10.1038/s41598-020-71536-3

**Published:** 2020-09-03

**Authors:** Shinsuke Nakazawa, Takatoshi Shimauchi, Atsuko Funakoshi, Masahiro Aoshima, Pawit Phadungsaksawasdi, Jun-ichi Sakabe, Sanki Asakawa, Noriyasu Hirasawa, Taisuke Ito, Yoshiki Tokura

**Affiliations:** 1 Department of Dermatology, Hamamatsu University School of Medicine, 1-20-1, Handayama, Higashi-Ku, Hamamatsu, 431-3192 Japan; 2 Laboratory of Pharmacotherapy of Life-Style Related Diseases, Graduate School of Pharmaceutical Sciences, Tohoku University, Sendai, Japan

**Keywords:** Experimental models of disease, Allergy, Atopic dermatitis

## Abstract

Suprabasin (SBSN) is expressed not only in epidermis but also in epithelial cells of the upper digestive tract where metals such as nickel are absorbed. We have recently shown that SBSN level is decreased in the *stratum corneum* and serum of atopic dermatitis (AD) patients, especially in intrinsic AD, which is characterized by metal allergy. By using SBSN-null (*Sbsn*^–/–^) mice, this study was conducted to investigate the outcome of SBSN deficiency in relation to AD. *Sbsn*^–/–^ mice exhibited skin barrier dysfunction on embryonic day 16.5, but after birth, their barrier function was not perturbed despite the presence of ultrastructural changes in *stratum corneum* and keratohyalin granules. *Sbsn*^–/–^ mice showed a comparable ovalbumin-specific skin immune response to wild type (WT) mice and rather lower contact hypersensitivity (CHS) responses to haptens than did WT mice. The blood nickel level after oral feeding of nickel was significantly higher in *Sbsn*^–/–^ mice than in WT mice, and CHS to nickel was elevated in *Sbsn*^–/–^ mice under nickel-loading condition. Our study suggests that the completely SBSN deficient mice retain normal barrier function, but harbor abnormal upper digestive tract epithelium that promotes nickel absorption and high CHS to nickel, sharing the features of intrinsic AD.

## Introduction

Suprabasin (SBSN) is a secreted protein that is expressed in the suprabasal layers of epithelia, including epidermis, tongue, esophagus, and forestomach in humans and mice^1–3^. SBSN is a substrate for transglutaminase 2 and 3 activity^2^, and knockdown of *Sbsn* downregulates cornified envelope (CE)-related genes in epidermal keratinocytes^4^. Meanwhile, overexpressed SBSN acts as an oncoprotein in esophageal squamous cell carcinoma by effecting on cell proliferation^5^. These findings suggest that SBSN plays a physiological role in the regulation of epidermal differentiation and skin barrier. However, the pathological significance of SBSN in barrier-damaged skin diseases is an issue to be elucidated.

Atopic dermatitis (AD) is an inflammatory disorder with skin barrier dysfunction and can be categorized into two types, serum IgE-high extrinsic AD, and serum IgE-normal intrinsic AD^6,7^. While extrinsic AD is the classical type with high prevalence, the incidence of intrinsic AD is approximately 20% of total AD with female predominance^6^. It is well known that extrinsic AD is caused by an impaired barrier function of the *stratum corneum*^6,8^*,* which is represented by the loss-of-function mutation of *filaggrin* (*FLG*)^9,10^. Extrinsic AD shows a Th2-skewing immunological condition as a consequence of protein antigen penetration through the impaired barrier and Langerhans cell operation as antigen-presenting cells^11^. On the other hand, the causes and mechanisms of intrinsic AD remain unclear. This subtype is immunologically characterized by high frequencies of circulating Th1 and Th17 cells in the peripheral blood^12^ and positive correlations between lesional skin Th17-related molecules and AD severity scores^13^. Non-protein antigens, such as metals, may induce dermatitis because intrinsic AD shows relatively normal barrier function^8^ and higher percentages of positive reactions to nickel and cobalt than extrinsic AD^6,14^. In addition, the serum nickel concentration is constitutionally high in intrinsic AD patients compared to extrinsic AD and healthy individuals^15^.

We have recently reported that amounts of SBSN in both *stratum corneum*^16^ and serum samples^17^ from AD patients are decreased compared to normal subjects. In particular, the serum SBSN level was lower in intrinsic AD than in extrinsic AD^17^. We also revealed that the expression of SBSN, unlike FLG, is unaffected by IL-4 and IL-13 in the epidermis. In SBSN-deficient 3-dimensional-reconstructed epidermis, there was abnormal epidermal differentiation, including compact *stratum corneum* and immature keratohyalin granules, independent of other differentiation markers, such as involucrin (IVL), claudin-1, and calpain-1^17^.

In this study, we created and analyzed SBSN-knockout (*Sbsn*^–/–^) mice to further clarify whether its deficiency leads to the AD-related conditions, including skin barrier function, skin responses to protein antigen and haptens, absorption of orally fed nickel, and contact hypersensitivity (CHS) to nickel. Results suggest the pathological significance of SBSN in intrinsic AD.

## Results

### Generation of Sbsn^–/–^ mice

**Figure 1:**
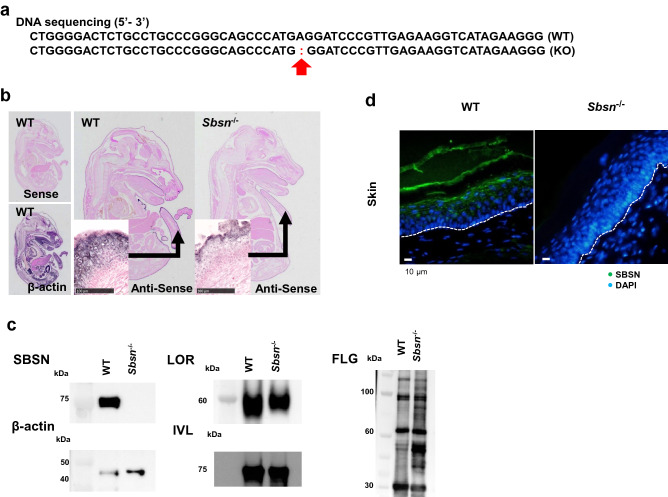
Characterization of *Sbsn*^–/–^ mice. (**a**) DNA sequence. The red arrow indicates the mutation location. (**b**) Whole-mount in situ hybridization of E16.5. In WT mice, SBSN mRNA was markedly expressed in the dorsal skin, tail skin, and oral epithelium, but it was not seen in KO mice. (**c**) Western blotting. The samples were RIPA buffer extracts from the epidermis. SBSN was completely knocked out in *Sbsn*^–/–^ mice. The expressions of LOR, IVL, and FLG were retained. The blots of SBSN and β-actin were cropped from one membrane. LOR, FLG, and IVL were cropped and reprobed from another membrane. (**d**) Immunofluorescence staining in footpad skin. Nuclei were counterstained with DAPI. SBSN (green) is observed in the upper epidermis and *stratum corneum* in WT, but not in *Sbsn*^–/–^ mice. The lines illustrate the basal membrane of epidermis.

*Sbsn* mutant heterozygote of C57BL/6 N mouse was generated by the CRISPR-Cas9 system. The mutation was detected in exon 1 (c.57delA, p.Glu26GlyfsTer8; Fig. 1a), the mutant was mated with C57BL/6NCr, and homozygous (KO, –/–) mice were established. In in situ hybridization, SBSN mRNA was markedly reduced in *Sbsn*-targeted mice (Fig. 1b). Western blotting analyses revealed that SBSN was completely deficient in *Sbsn*^–/–^ mice, whereas other epidermal differentiation markers, loricrin (LOR), IVL and FLG, were unaffected (Fig. 1c). Immunohistochemically, SBSN was absent in the epidermis of *Sbsn*^–/–^ mice (Fig. 1d). *Sbsn*^–/–^ mice were viable and healthy, without apparent growth restriction.

### SBSN deficiency affects skin barrier at embryo, but not after birth

**Figure 2:**
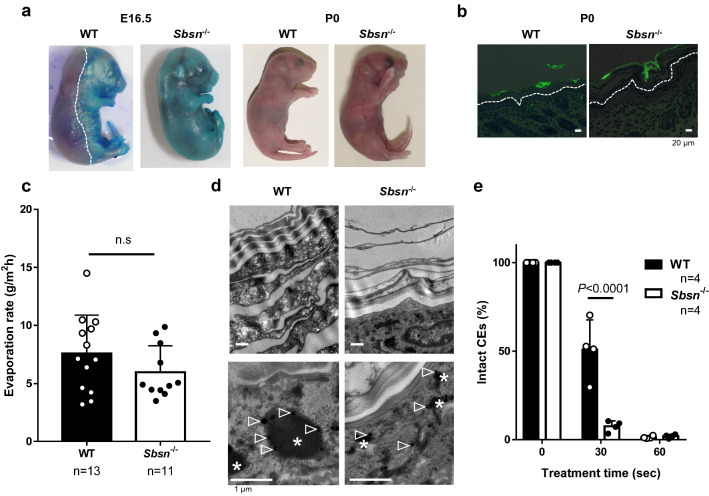
Skin barrier function of *Sbsn*^–/–^ mice. (**a**) Toluidine Blue staining. Left and right panels are E16.5 and newborn (P0), respectively. The line indicates the edge of the functional skin barrier. (**b**) Lucifer-Yellow penetration. Dorsal skin samples of the newborn were immersed in 1 mM lucifer yellow solution for 1 h. The lines indicate the basal membrane of epidermis. (**c**) Transepidermal water loss (TEWL). The inside-outside barrier function was assessed by TEWL. No difference is observed between WT and *Sbsn*^–/–^ mice. (**d**) Ultrastructural analysis (TEM). Upper panel, *stratum corneum*; and lower panel, keratohyalin granules. *, keratohyalin granule; and arrowheads, ribosomes. (**e**) Fragility of cornified envelopes (CEs). CEs were sonicated for the indicated time. CEs isolated from *Sbsn*^–/–^ mice were more rapidly destroyed by sonication than WT mice as indicated by the percentage of intact CEs.

In WT mice, toluidine blue was not penetrated through the dorsal skin at E16.5, indicating the development of barrier at this embryonic day. In *Sbsn*^–/–^ embryo, the dorsal skin allowed toluidine blue to penetrate the barrier (Fig. 2a, left panel). However, newborn *Sbsn*^–/–^ mice (P0), similarly to WT mice, did not have the outside-inside barrier dysfunction (Fig. 2a, right panel). Moreover, no penetration of lucifer yellow across the *stratum corneum* was observed in either WT or *Sbsn*^–/–^ mice (Fig. 2b), confirming that skin barrier was not perturbed in newborn *Sbsn*^–/–^ mice. When the inside-outside barrier was assessed by transepidermal water loss (TEWL), there was no difference between adult WT and 7-week old *Sbsn*^–/–^ mice (Fig. 2c).

Skin ultrastructure was assessed using transmission electron microscopy (TEM). Whereas the *stratum corneum* of WT newborn mice was thick and filled with dense materials, that of *Sbsn*^–/–^ mice was scarce (Fig. 2d, upper panel). Keratohyalin granules (*) of *Sbsn*^–/–^ mice were immature, which contained profilaggrin and LOR (Fig. 2d, lower panel; arrowheads, ribosome). To examine whether the thin *stratum corneum* is associated with CE abnormality, the fragility of CEs isolated from the newborn mouse skin was evaluated. CEs from *Sbsn*^–/–^ were more rapidly destroyed by sonication than those from WT mice (Fig. 2e). Thus, despite these ultrastructural abnormalities, neither outside-inside nor inside-outside barrier dysfunction was detected in newborn *Sbsn*^–/–^ mice. The *stratum corneum* and keratohyalin granules of 8-week-old *Sbsn*^–/–^ mice had the same ultrastructural changes as those of newborns. The fragility of CEs in the older mice was also comparable to the newborns. Thus, the older mice did not show critical barrier dysfunction either.

### *Sbsn*^–/–^ mice have normal ovalbumin (OVA)-specific IgE response and rather low CHS responses to haptens

**Figure 3:**
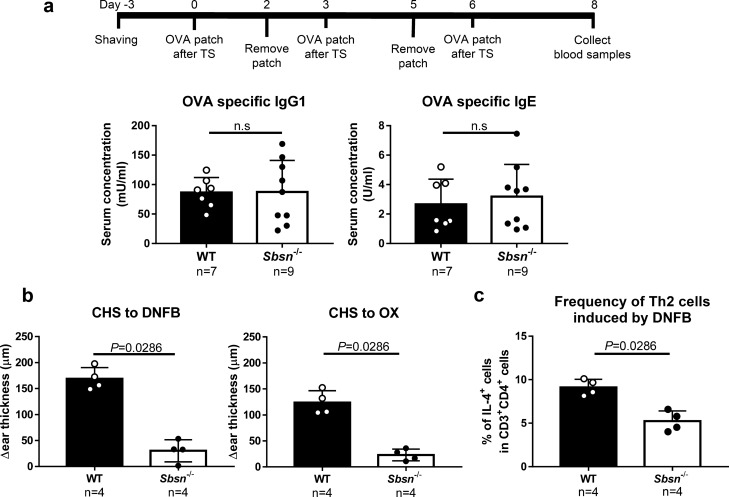
Transcutaneous immunization to protein antigen in *Sbsn*^–/–^ mice. (**a**) OVA-specific immunoglobulins. TS, tape stripping. Trans-cutaneous immunization response was performed in mice. Any statistically differents were observed. (**b**) CHS response to DNFB and OX. *Sbsn*^–/–^ mice show rather lower ear swelling responses to DNFB and oxazolone than did WT mice. (**c**) Frequency Th2 cells induced by DNFB. The frequency of IL-4^+^CD4^+^ T cells is significantly lower in *Sbsn*^–/–^ mice than in WT mice.

The transcutaneous immunization of OVA was evaluated in *Sbsn*^–/–^ along with WT mice (Fig. 3a). There was no difference between WT and *Sbsn*^–/–^ mice in either OVA-specific IgG1 or IgE production. Thus, transcutaneous immunization for protein antigen was not promoted by the SBSN deficient condition.

When CHS responses to haptens were tested, *Sbsn*^–/–^ mice unexpectedly showed rather lower ear swelling responses to 2,4-dinitro-1-fluorobenzene (DNFB) and oxazolone than did WT mice (Fig. 3b). We therefore addressed the background for the low hapten response of *Sbsn*^–/–^ mice. The phenotypical abnormality was not found in peripheral T cell populations in *Sbsn*^–/–^ mice (data not shown). We then measured the percentage of IFN-γ^+^CD4^+^, IL-4^+^CD4^+^, and IL-17A^+^CD4^+^ T cells in the lymph nodes taken from DNFB-sensitized mice by flow cytometry. There was no significant difference in the frequency of IFN-γ^+^CD4^+^ or IL-17A^+^CD4^+^ T cells between *Sbsn*^–/–^ and WT mice (data not shown). However, the frequency of IL-4^+^CD4^+^ T cells was significantly lower in *Sbsn*^–/–^ mice than in WT mice (Fig. 3c). Thus, the Th1/Th2 balance was skewed relatively to Th1 cells in *Sbsn*^–/–^ mice, mimicking intrinsic AD. There was no difference in the Langerhans cell number and its migratory ability to draining lymph nodes between *Sbsn*^–/–^ and WT mice (data not shown). Therefore, the low CHS of *Sbsn*^–/–^ mice seems to stem from an as-yet-uncharacterized systemic immune abnormality.

### Blood nickel level is high in Sbsn^–/–^ mice after oral nickel loading

**Figure 4:**
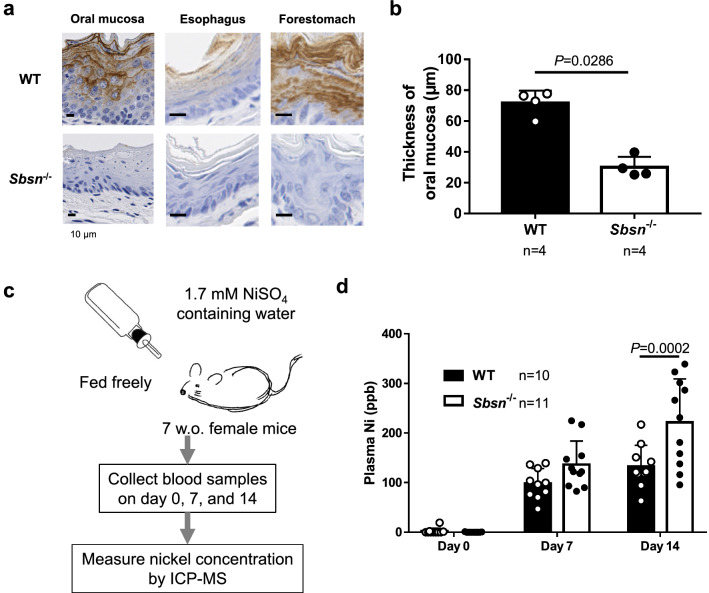
Blood nickel level in orally nickel-loaded *Sbsn*^–/–^ mice. (**a**) Immunohistochemical staining in upper digestive tracts. Oral mucosa, esophagus, and forestomach were positively stained with anti-SBSN antibody in WT mice, but not in *Sbsn*^–/–^ mice. Note that the epithelia of *Sbsn*^–/–^ mice were thinner than those of WT mice. (**b**) Thickness of oral mucosa. The oral mucosa of *Sbsn*^–/–^ mice was thinner than in WT. (**c**) Procedure of nickel loading test. The plasma nickel level was measured on day 0, 7, and 14. (**d)** Blood nickel level. The blood nickel level after oral feeding of nickel was higher in *Sbsn*^–/–^ mice than in WT mice on day 14.

SBSN expression of *Sbsn*^–/–^ mice was completely absent not only in the skin but also in the upper digestive tract, including oral mucosa, esophagus, and forestomach (Fig. 4a). Notably, the mucosal thickness of *Sbsn*^–/–^ mice was thinner than that of WT mice (Fig. 4b). In the oral mucosa, esophagus, and skin of *Sbsn*^–/–^ mice, there was no inflammation in a steady state.

In addition to duodenum, upper digestive tract is an important site for the absorption of metals^18–20^. In conventional housing of mice, the blood nickel concentration was under detection level in both WT and *Sbsn*^–/–^ mice. To evaluate the nickel absorption, we fed 100 ppm nickel water (1.7 mM NiSO_4_) freely to mice and collected blood samples on day 0, 7, and 14 (Fig. 4c). The plasma nickel concentration was higher in *Sbsn*^–/–^ mice than in WT mice on day14 (Fig. 4d). Thus, nickel absorption was increased presumably in the upper digestive tract epithelial cells of *Sbsn*^–/–^ mice. Under nickel loading, neither WT nor *Sbsn*^–/–^ mice suffered from anaphylactic symptoms such as diarrhea.

### CHS to nickel is promoted under nickel loading condition in *Sbsn*^–/–^ mice

**Figure 5:**
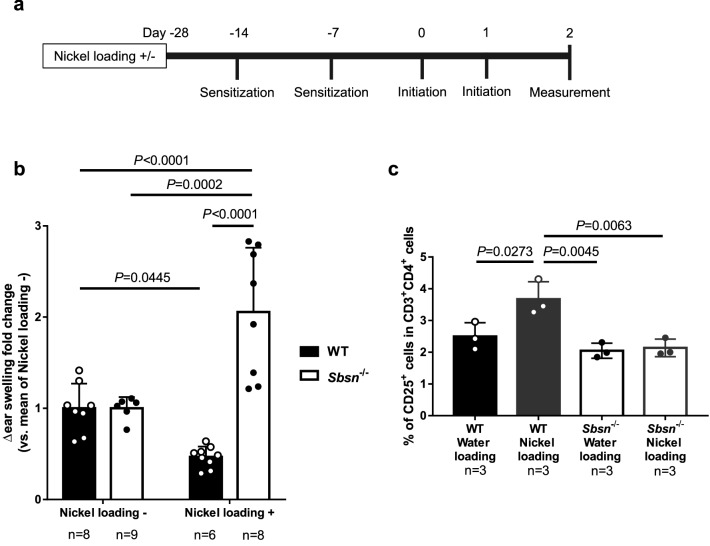
CHS responses to nickel in nickel-loaded mice. (**a**) Time course of CHS to nickel in nickel-loaded mice. Mice were fed with 100 ppm nickel (1.7 mM NiSO_4_) or normal water 4 weeks before, and they were sensitized and elicited as mentioned. (**b**) Ear swelling responses in nickel- or water-loaded mice sensitized and challenged with nickel. Nickel loading decreased CHS response in WT mice. *Sbsn*^–/–^ mice showed augmented ear swelling by nickel loading. (**c**) Frequency of CD4^+^CD25^+^ T cells with or without nickel loading. While drinking of low-dose nickel-containing water increases CD4^+^CD25^+^ T cells in WT, it unaffected their frequency in *Sbsn*^–/–^ mice.

Metal allergy, especially to nickel, cobalt, and chromium, often coexists in AD patients^15,21^. When AD patients take in excessive metal, their eczema is occasionally exacerbated^6^. Since the blood nickel level was high in *Sbsn*^–/–^ mice, it is an interesting issue whether CHS to nickel is altered in nickel-loading *Sbsn*^–/–^ mice. To test the effect of nickel loading, the CHS to nickel was examined, according to the reported protocol^22,23^ with some modifications (Fig. 5a). Ear swelling was measured under continuous water loading or non-loading condition. The oral nickel loading per se did not induce the ear swelling in a comparison between the data on day -14 and day 0. Without nickel loading, the CHS magnitude to nickel was detectable in *Sbsn*^–/–^ mice (Δ ear thickness, ~ 30 µm), but similarly to the hapten responses, lower than that in WT mice (Δ ear thickness, ~ 115 µm). Therefore, to compare the effect of nickel loading between WT and *Sbsn*^–/–^ mic, the levels of ear swelling responses to nickel in both nickel-non-loading WT and *Sbsn*^–/–^ mice were regarded as “1”, and the fold changes were calculated in the nickel-loading mice (Fig. 5b). The continuous nickel loading suppressed the swelling response of WT mice (closed bars), possibly by oral tolerance. On the contrary, the nickel loading significantly augmented the response of *Sbsn*^–/–^ mice (open bars). Thus, *Sbsn*^–/–^ mice showed a markedly enhanced ear swelling response to nickel under the nickel loading condition.

It has been suggested that oral intake of low dose nickel induces tolerance, although this phenomenon remains unclear. To address the possibility that oral tolerance is attenuated in *Sbsn*^–/–^ mice, we examined the percentage of regulatory T (Treg) cells in splenic T cells. We analyzed splenic T cells from mice orally given nickel for 2 weeks by flow cytometry. The percentage of CD25^+^ cells in CD4^+^ T cells was calculated. While drinking of low-dose nickel-containing water increased CD4^+^CD25^+^ T cells in WT mice, it unaffected their frequency in *Sbsn*^–/–^ mice (Fig. 5c). The results suggest that oral tolerance did not operate in *Sbsn*^–/–^ mice.

## Discussion

We have demonstrated that the amount of SBSN is decreased in the *stratum corneum* and the sera of AD patients compared to normal subjects^16,17^. Another group of investigators also reported that SBSN deficiency is one of the factors related to AD pathogenesis^4^. Similarly to knockout mice for the other epidermal differentiation-associated proteins^24–27^, *Sbsn*^–/–^ mice were viable and healthy, without apparent growth restriction. *Sbsn*^–/–^ mice did not show any of the crucial features of typical (extrinsic) AD, including skin barrier dysfunction and high transcutaneous responses. Only the embryonic phase, *Sbsn*^–/–^ embryo had skin barrier dysfunction. After birth, the dysfunction was not seen despite ultrastructural abnormalities. Such a phenomenon was also observed in *Lor*^–/–^ mice^25^. SBSN deficiency did not affect the expression of LOR, IVL, or FLG. Similarly, proteomic analysis of FLG deficiency showed that SBSN is independent of FLG in its expression^28^. In our *Sbsn*^–/–^ mice, SBSN deficiency led to the ultrastructural difference in the *stratum corneum* and keratohyalin granules, and CEs fragility. These ultrastructural features were also observed in our study using *Sbsn* knockdown human epidermal keratinocytes^17^. In another study using a culture system of *Sbsn* knockdown mouse keratinocytes, genes for CE, including late cornified envelope and small proline-rich protein, were down-regulated^4^. Although CEs were fragile in our mice, skin barrier function was maintained after birth, similarly to *Lor*^–/–^ mice^25,29^. Our findings suggest that SBSN did not exert a profound effect on the barrier ability of the *stratum corneum*.

Upon oral feeding of nickel, the blood nickel level in *Sbsn*^–/–^ mice was higher than in WT mice. SBSN is expressed not only in the skin but also in the oral mucosa, tongue, and esophagus^1^. It was reported that mRNAs for epithelial lining cytokeratins, *Krt 6b, 7, 18 and 19,* were down-regulated in *Sbsn* knockdown^4^. We consider that SBSN shortage in epithelia possibly contributes to increased metal absorption and resultant elevation of blood nickel concentration. In this concept, we found that the thickness of oral mucosa in *Sbsn*^–/–^ mice was significantly lower than in WT.

Since intrinsic AD patients show high serum nickel concentration^14^, high frequency of positive nickel patch test^13^, and occasional exacerbation of AD by nickel intake^15,21^, we investigated the cutaneous allergic reaction to nickel in *Sbsn*^–/–^ mice. The CHS response to nickel was remarkably augmented by oral nickel loading in *Sbsn*^–/–^ mice. While the dose dependence of oral nickel tolerance was reported in C57BL/6^30–34^, as seen in our WT mice, our finding is consistent with the above clinical notion, reflecting the condition of intrinsic AD.

Intrinsic AD is characterized by preserved skin barrier, low serum IgE level, Th1/Th17-skewing immune condition, high blood nickel level, and high coincidence of nickel allergy^6,14,15^. Notably, we assured that the serum SBSN level was lower in intrinsic than extrinsic AD^17^. In addition to the preserved skin barrier function, *Sbsn*^–/–^ mice presented with a normal IgE response to OVA and a high blood nickel level upon oral loading. In contrast, *Flaky tail, Flg*^*–/–*^ and *Tmem79*^*ma/ma*^ mice have skin barrier dysfunction that induces intense allergen-specific immune responses^24,35–38^. Given that FLG deficiency is a representative feature of extrinsic AD, SBSN deficiency may provide a non-extrinsic property. We found that oral absorption of nickel is abnormally upregulated in intrinsic AD patients^15^.

We demonstrated that nickel fed mice showed increased blood nickel levels. It strongly suggests that the absence of SBSN in the upper digestive tract epithelia caused the high blood nickel level and the increased CHS responses to nickel. Since newborn and adult *Sbsn*^–/–^ mice retained the skin barrier function toward toluidine blue, lucifer yellow, and OVA, enhancement of nickel penetration through the skin of *Sbsn*^–/–^ mice is unlikely. Therefore, the increased CHS responses to nickel seem to be attributable to the impaired upper digestive tract epithelia and resultant high nickel absorption. However, we cannot completely exclude a possibility that the absence of SBSN in the epidermis increased the efficiency of penetration of nickel via the skin, leading to the increased CHS responses. Moreover, we proposed the anti-apoptotic effect of SBSN on epidermal keratinocytes^15^. The absence of this anti-apoptotic action may lead to the fragility of the epithelia, allowing nickel to be increasingly absorbed in *Sbsn*^–/–^ mice.

Unexpectedly, the ear swelling responses to haptens and nickel were low in *Sbsn*^–/–^ mice compared to WT mice. We investigated the factors seemingly responsible for this low responsiveness, such as T cell phenotypes and Langerhans cell distribution and migration, but currently, we could not detect differences between *Sbsn*^–/–^ and WT mice. The only difference that we found is a low frequency of IL-4-producing Th2 cells in the draining lymph nodes of DNFB-sensitized *Sbsn*^–/–^ mice, which might be associated with the low CHS responses. We consider that *Sbsn*^–/–^ mice have an as-yet-unknown systemic immune abnormality presumably in SBSN-expressing organs or cells.

Nevertheless, the CHS response to nickel was remarkably augmented by oral nickel loading in *Sbsn*^–/–^ mice, whereas it was decreased by nickel feeding in WT mice. Given the general concept of oral tolerance, the enhancement of nickel CHS by oral nickel, seen in *Sbsn*^–/–^ mice, is peculiar. The relationship between high blood nickel level and nickel allergy is speculative in both human intrinsic AD and our mouse model. Treg cells are usually induced by oral nickel^33^. We found that the number of CD4^+^CD25^+^ T cells was increased by nickel loading in WT mice, but it was unchanged in *Sbsn*^–/–^ mice. The number of CD4^+^CD25^+^ T cells was lower in systemic nickel hypersensitivity patients than in healthy control^33,39–41^. Given that intrinsic AD shares the features with systemic nickel hypersensitivity, orally tolerogenic action of nickel might be attenuated in intrinsic AD.

Our study suggests that SBSN is involved in not only epidermal differentiation but also absorption and immunity of nickel in the upper digestive tract. Its deficient condition in human intrinsic AD is an issue to be further elucidated in the future.

## Materials and methods

### Generation of Sbsn^–/–^ mice using the CRISPR-Cas9 system

*Sbsn* is located on chromosome 7 in *Mus musculus.* The C57BL/6N heterozygous were obtained by using the CRISPR-Cas9 technique services of TaKaRa (Shiga, Japan), as described in detail in Supplementary Materials and Methods online (Suppl 1,2). The mutation was detected in exon 1 (c.57delA), the mutant was mated with C57BL/6NCr, and we established homozygous (KO, –/–) mice.

### Mice

WT mice (C57BL/6NCr) were purchased from Japan SLC (Shizuoka, Japan). Six to ten-week-old female WT and KO mice were used to study adult mice, and neonatal mice within 24 h of birth were used to study newborn mice. All procedures were performed in accordance with the guidelines for proper conduct of animal experiments stated by the Science Council of Japan and the protocols approved by the Hamamatsu University School of Medicine Ethics Committee for Animal Experiments.

### In situ hybridization

Mouse E16.5 was fixed with G-Fix (Genostaff, Tokyo, Japan), embedded in paraffin and sectioned at 8 μm. Whole-mount in situ hybridization was performed by Genostaff (Tokyo, Japan), as described in detail in Supplementary Materials and Methods online (Suppl 2).

### Western blotting analysis

The dorsal skin was obtained from freshly sacrificed WT and KO newborn mice as described in detail in Supplementary Materials and Methods online (Suppl 3). Blots were reacted with antibodies to suprabasin (Eurofins Genomics K. K., Tokyo, Japan), filaggrin, loricrin, involucrin (all from BioLegend, San Diego, CA), and β-actin (Cell Signaling Technology, Inc., Beverly, MA). Immunoreactive proteins were visualized with the chemiluminescent substrates for horseradish peroxidase. Suprabasin antibody recognized the following peptide: REVEKIFGELSNMGSQAGKNVEHGLDKVAHD. The full-length blots were included in Supplementary Materials and Methods online (Suppl 4).

### Toluidine blue staining

The development of epidermal barrier was analyzed by dye penetration assay as described by Koch et al.^25^. Briefly, the embryos/newborns were dehydrated by series of 1 min-incubation in 25%, 50%, 75% methanol/PBS and 100% methanol, then rehydrated with the same series of methanol solutions and washed in PBS. The embryos were stained for 1 min, the newborns were stained 5 min in 0.0125% toluidine blue/PBS.

### Lucifer yellow staining

The dorsal skin of the newborn mouse was immersed in 1 mM lucifer yellow CH dilithium (Santa Cruz Biotechnology, Santa Cruz, Calif) for 1 h after which the mice were killed. Paraffin sections, penetration of the dye were assessed by immunofluorescence microscopy, as described by Koch et al.^25^.

### TEWL

The dorsal hair of the mouse was removed with an electric shaver. One day after the shaving, TEWL was measured using a tewameter TM 300 (Courage & Khazaka electronic, Cologne, Germany) as described in detail in Supplementary Materials and Methods online (Suppl 5).

### Electron microscopy

Transmission electron microscopy was performed on the dorsal skin of KO and WT newborn mice. Skin samples were fixed with 2% glutaraldehyde and 1% osmium tetroxide and processed for conventional TEM (JEM1400: JEOL, Tokyo, Japan) as described in detail in Supplementary Materials and Methods online (Suppl 6).

### Isolation and analysis of cornified envelopes

Leclerc’s cornified envelopes fragility assay was used^26^. The newborn mouse skin was boiled for 10 min at 95 °C under vigorous agitation in cornified envelope (CE) isolation buffer (100 mM Tris–HCl, 20 mM DTT, 5 mM EDTA) containing 2% SDS. CEs were centrifuged at 12,000 g and resuspended in CE isolation buffer containing 2% SDS. Purified CEs were then washed 3 times in isolation buffer containing 0.2% SDS. The CE suspension (2 × 10^5^/mL) was sonicated for 30 and 60 s. Intact and fragile CEs were counted using a hemocytometer.

### OVA sensitization

OVA sensitization test was performed as described by Nakajima et al.^11^. Briefly, a shaved back skin was tape-stripped 5 times with adhesive cellophane tape (Nichiban, Tokyo, Japan). Each mouse had a total of three 2-day exposures to the OVA patch (1 μg/ml, Torii Pharmaceutical, Co, Ltd, Tokyo, Japan), separated by 1-day intervals. Mice were euthanized at the end of the third cycle of sensitization. OVA-specific serum IgG_1_ and IgE levels were detected 3 days after the final application using commercial ELISA kits (Shibayagi, Shibukawa, Gunma, Japan) following the manufacturer's instructions.

### Sensitization and elicitation of CHS

2,4-dinitro-1-fluorobenzene (DNFB) was dissolved in 4:1 (vol/vol) acetone/olive oil. Mice were sensitized with 0.5% DNFB by application to the shaved abdomen (25 μl) on day 0. They were challenged on the right ear on day 7 with 20 μl of 0.3% DNFB. Oxazolone (OX) was dissolved in acetone. Mice were sensitized with 1.5% OX by application to the shaved abdomens (100 μl) on day 0. They were challenged on the right ear on day 7 with 20 μl of 0.5% OX. Ear thickness was measured using a digital micrometer (Mitutoyo, Kawasaki, Japan). Ear thickness change was calculated as (ear thickness 24 h after challenge)-(ear thickness before challenge).

### Flow cytometry

For Th1/Th2 cytokine analysis, bilateral groin lymph nodes were harvested at 3 days after sensitization by DNFB. Cells were then activated using 25 ng/ml of Phorbol 12-Myristate 13 Acetate and 1 μg/ml of ionomycin with GolgiStop (BD Biosciences, San Diego, CA) in culture medium for 4 h. After activation, cells were stained with anti-CD4 allophycocyanin (APC) (clone GK1.5, BioLegend), anti-CD3 APC cyanin 7 (Cy7) (clone 17A2, BioLegend). Intracellular staining was performed using fixation and permeabilization kit (BD Bioscience) with anti-IFN-γ fluorescein isothiocyanate (FITC) (clone XMG1.2, BioLegend) and anti-IL-4 peridinin chlorophyll protein (PerCP) (clone 11B11, BioLegend). For determination of CD4^+^CD25^+^ T cells, mice were fed with 100 ppm of nickel water freely for two weeks, and then, splenic single-cell suspensions were prepared using BD lysing buffer (BD Bioscience). Cells were incubated with anti-CD16/CD32 mAb for Fc blocking (clone 2.4G2, BD Bioscience), anti-CD3 FITC (clone 17A2, BioLegend), anti-CD25 phycoerythrin (PE) (clone 3C7, BD Bioscience), and anti-CD4 APC (GK1.5, BioLegend). Cells were acquired on a FACS CantoII (BD Biosciences). Data were analyzed with FlowJo v10 software (FlowJo LLC, Ashland, OR).

### Immunohistochemical and immunofluorescence staining for SBSN

Tissues were fixed in 10% buffered formalin and embedded in paraffin. For frozen sections, footpad samples were embedded in Tissue-Tek OCT compound (Sakura Finetechnical, Tokyo, Japan) in liquid nitrogen. 5 μg/ml of rabbit anti-mouse SBSN antibody (Eurofins Genomics K. K.) was incubated. Secondary antibodies were Horse Radish Peroxidase (HRP) labeled and Alexa Fluor 488 labeled goat anti-rabbit IgG (Nichirei, Tokyo, Japan, and Thermo Fisher Scientific, Waltham, MA). Different 5 sights of oral mucosa taken from the cheek were measured using NDP.view2 (Hamamatsu Photonics KK, Hamamatsu, Japan). The average thickness of each mouse was shown.

### Nickel loading test

Mice drank water containing additional levels of 100 ppm nickel (1.7 mM NiSO_4_) freely. Blood samples were collected on day 0, 7, and 14. Plasma Nickel densities were determined by inductively coupled plasma mass spectrometry (Mitsubishi Chemical Analytech, Tokyo, Japan).

### Sensitization and elicitation of CHS to nickel with or without nickel loading

We used the sensitization and elicitation protocol to nickel reported by Kinbara et al.^23^ and Vennegaard et al.^22^. Mice drank 100 ppm nickel (1.7 mM NiSO_4_) water or normal water. Two weeks later, a total of 125 µl of 1 mM NiCl_2_ with 1 µg/ml LPS in saline was injected twice into the left and right groin of mice at an interval of 7 days via the intradermal (id) route (250 µl per mouse). Further 7 days later, CHS response was elicited by skin application of 10% NiCl_2_ in white petrolatum for 24 h to inner pinna (15–20 mg per ear). Ear thickness was measured under continuous water loading conditions. The net swelling (i.e. the difference between pre- and post-challenge) was recorded. To compare the effect of nickel loading between *Sbsn*^–/–^ and WT mice, the fold change was calculated as: net swelling of nickel loading / mean net swelling of normal water loading.

### Statistical analysis

Experiments were analyzed by using the Mann–Whitney's U test and ordinary one-way ANOVA with Sidak’s multiple comparisons test with GraphPad Prism 7.0 software (GraphPad Software, Inc, San Diego, Calif). In Fig. 5b,c, the result was analyzed by the two way ANOVA with Turkey’s multiple comparisons test. All results are presented as means ± SD. A *P* value of less than 0.05 was considered significant.

## Supplementary information

Supplementary Information.
