# Supplementary material for: Suprabasin-null mice retain skin barrier function and show high contact hypersensitivity to nickel upon oral nickel loading
Source: Sci Rep. 2020 Sep 3;10:14559. doi: 10.1038/s41598-020-71536-3 (PMC7471289; doi:10.1038/s41598-020-71536-3)
Supplement: Supplementary file 1 — Supplementary Information. [file 41598_2020_71536_MOESM1_ESM.docx]

**Title:**

**Suprabasin-null mice retain skin barrier function and show high contact hypersensitivity to nickel upon oral nickel loading**

**Authors:**

Shinsuke Nakazawa, MD,^1^ Takatoshi Shimauchi, MD, Ph.D,^1^ Atsuko Funakoshi, Ph.D,^1^ Masahiro Aoshima, MD,^1^ Pawit Phadungsaksawasdi, MD,^1^ Jun-ichi Sakabe, Ph.D,^1^ Sanki Asakawa, Ph.D,^2^ Noriyasu Hirasawa, Ph.D,^2^ Taisuke Ito, MD, Ph.D,^1^ and *Yoshiki Tokura, MD, Ph.D,^1^

**Correspondence:** Yoshiki Tokura MD Ph.D.,

Department of Dermatology, Hamamatsu University School of Medicine, 1-20-1

Handayama, Higashi-Ku, Hamamatsu 431-3192, Japan

E-mail: tokura@hama-med.ac.jp

Phone: +81-53-435-2303, Fax: +81-53-435-2368

**Affiliations**

^1^Department of Dermatology, Hamamatsu University School of Medicine, Hamamatsu, Japan

^2^Laboratory of Pharmacotherapy of Life-style Related Diseases, Graduate School of Pharmaceutical Sciences, Tohoku University, Sendai, Japan

**SUPPLEMENTARY MATERIAL AND METHOD**

**Suppl 1**

**Generation of *Sbsn*^-/-^ mice**

*Sbsn*^-/-^ mice were generated using the CRISPR-Cas9 genome-editing system and were maintained on the C57BL/6N background. The target sequence of Cas9/sgRNA were designed from following sequence: 5’-CCCATGAGGATCCCGTTGAGAAG-3’. The genotype of *Sbsn* and/or a null allele was determined by direct sequencing of amplified genomic DNA with following primers; F: 5’-agcctcacaggagggattga-3’, R: 5’-tccgatgccactgttgatgt-3’. The *in situ* hybridization probe sequence position was 172-422 of ENSMUSG00000046056 (5’-TCCCGTTGAGAAGG-GAAGTTTGCCCATG-3’).

**Suppl 2**

**Genomic Sequences**

Ensembl: ENSMUSG00000046056. Showing position:-50 to 500

-50 acacccccttgactgaaccgaagtcagagcctcacaggagggattgagtc

1 atgaggggtggggaagagggagggaggtgaaggataaatagcggcctcgc tccctggctcctctctgcatcctccctgccttcccaacaacatgtatctt gtcagtttgctcagctcctgctgcctcttagtgctcctggggactctgcc tgcccgggcag**cccatgaggatcccgttgagaag**gtcatagaagggttca gccgagggctgagcaatgctgagagagaggtgggcaaggcccttgaaggc atcaataacggaatcactcaagctggaagggaagtggaaaaaatttttgg tgaactcagcaacatgggcagccaggctggcaagaacgtggaacatggct tggacaaagtagcccacgacatcaacagtggcatcggacacgcaggaaag gaagcagagaagtttgcccatggggtcaaccacgccgctggacaggttgg gaaggagacaaacaaaatcatccatcatggggtcagccaggggggcagtg

yellow: F primer sequence

bold: target sequence for sgRNA

green: R primer sequence

under bar: the probe sequence for *in situ* hybridization

**Suppl 3**

**Western blotting analysis**

The epidermis was separated from the dermis by immersion in PBS containing 5 mM EDTA at 50°C for 2 min, followed by rapid cooling in cold PBS. The separated dorsal epidermis was extracted in 150 μl of RIPA buffer containing protease inhibitors with a homogenizer (BioMasher; Nippi, Tokyo, Japan). The samples were sonicated for 20 minutes. For immunoblotting, equal protein loadings were separated by SDS-PAGE on NuPAGE 4-12% w/v Bis-Tris gels (Invitrogen, Carlsbad, CA) at 200 V, and electroblotted for 2h at 0.4A on to nitrocellulose membrane.

**Suppl 4**

**The full-length blots**

Two membranes were separated from one gel and were reprobed using the stripping solution (Wako, Osaka, Japan). The blots of SBSN and β-actin were cropped from one membrane. LOR, FLG, and IVL were reprobed from another membrane.

SBSN


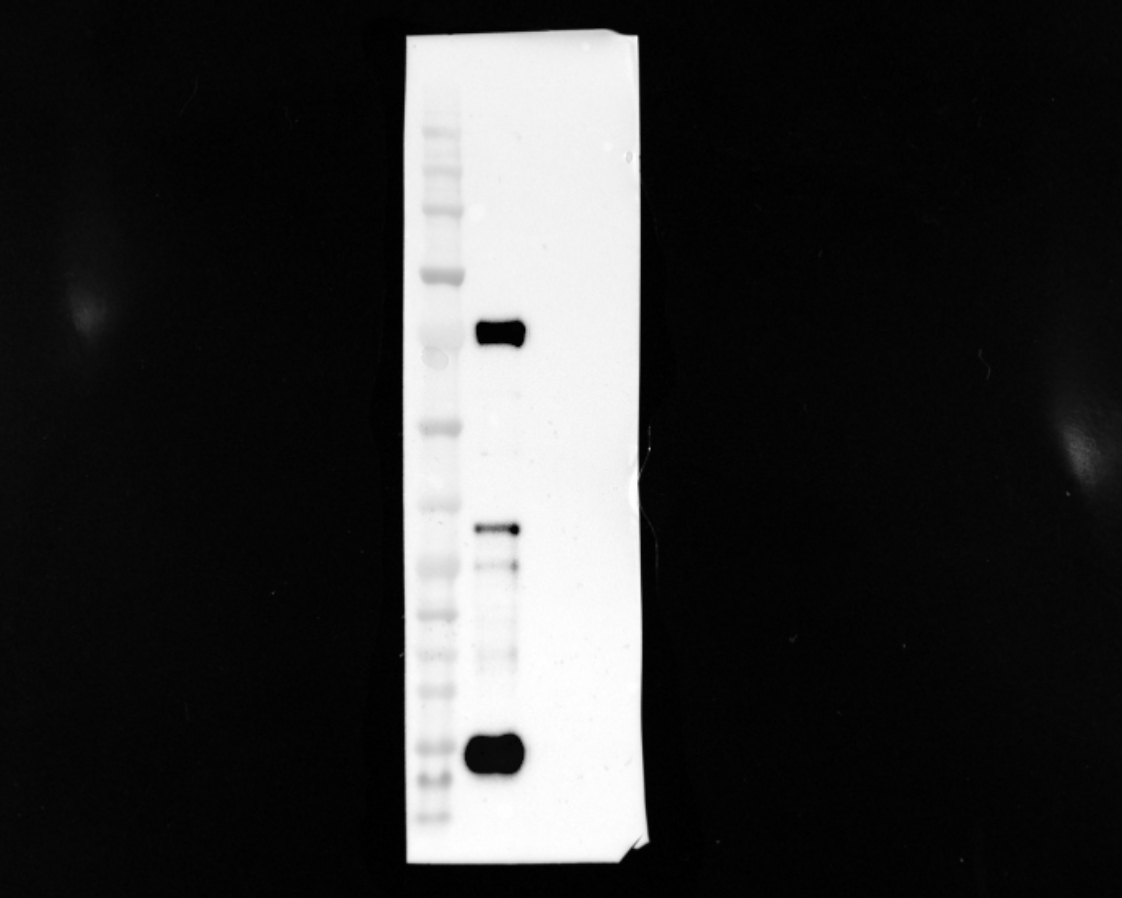


β-actin
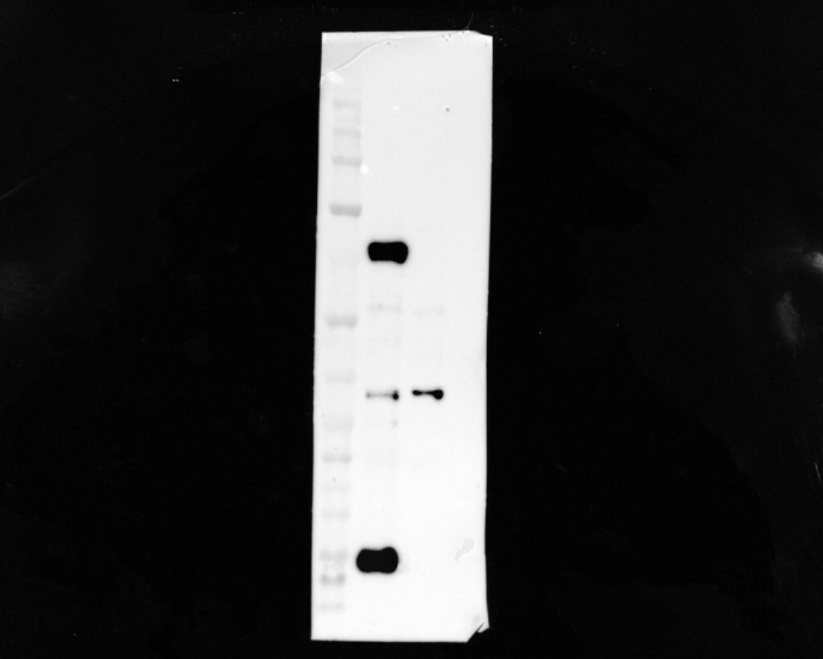


LOR


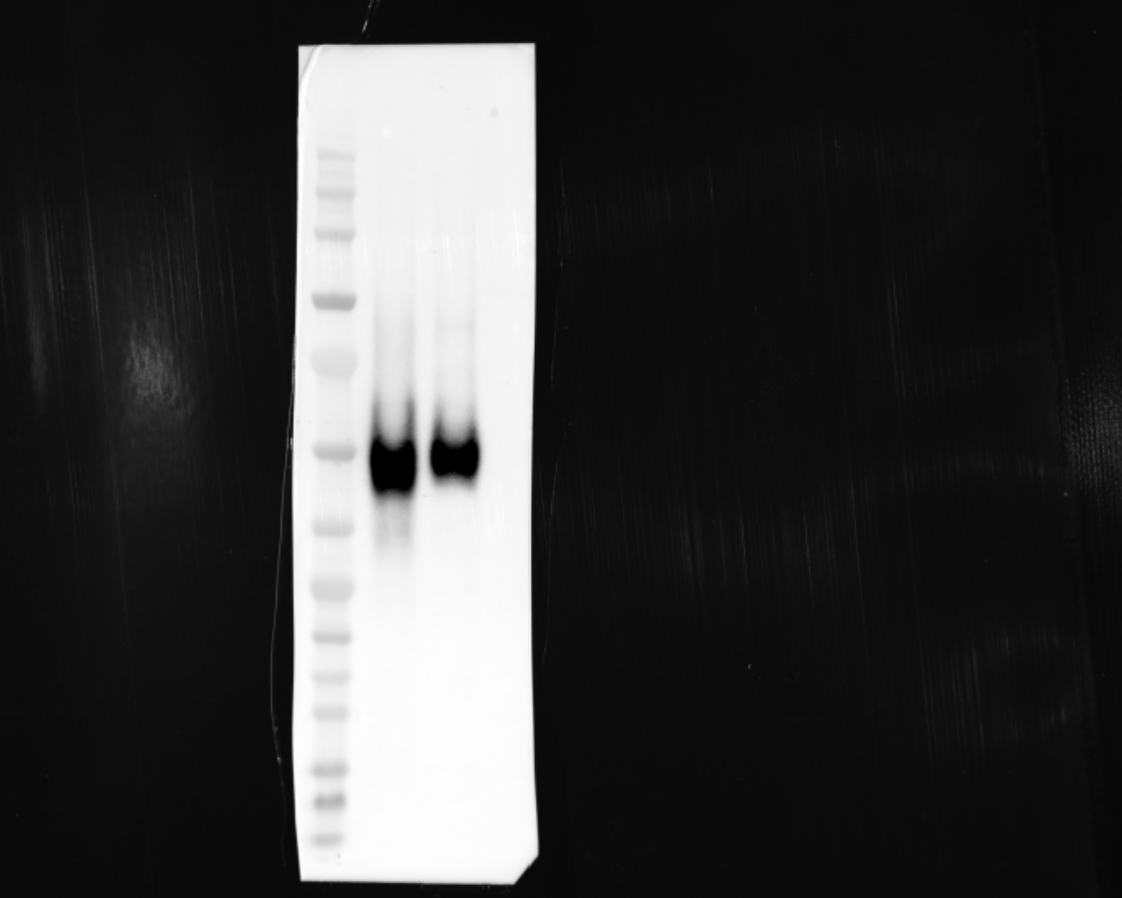


FLG


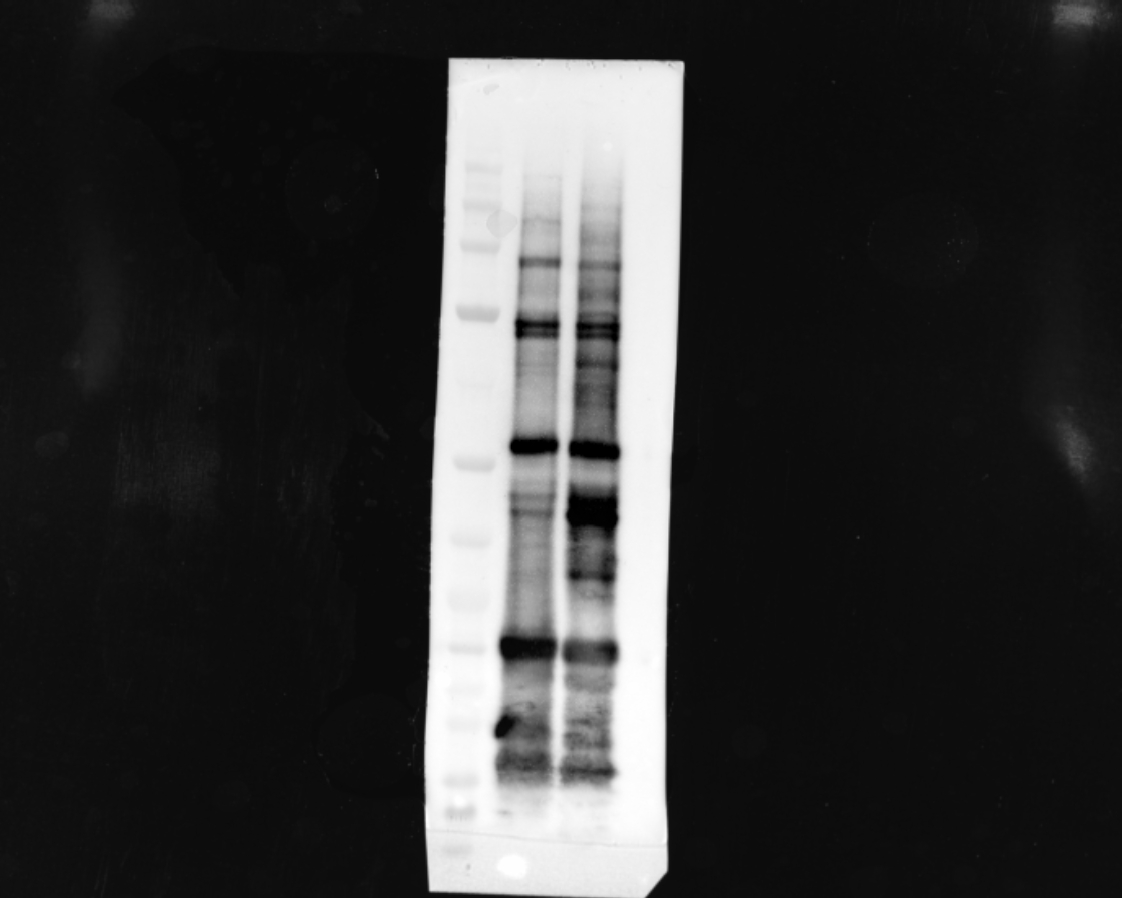


IVL


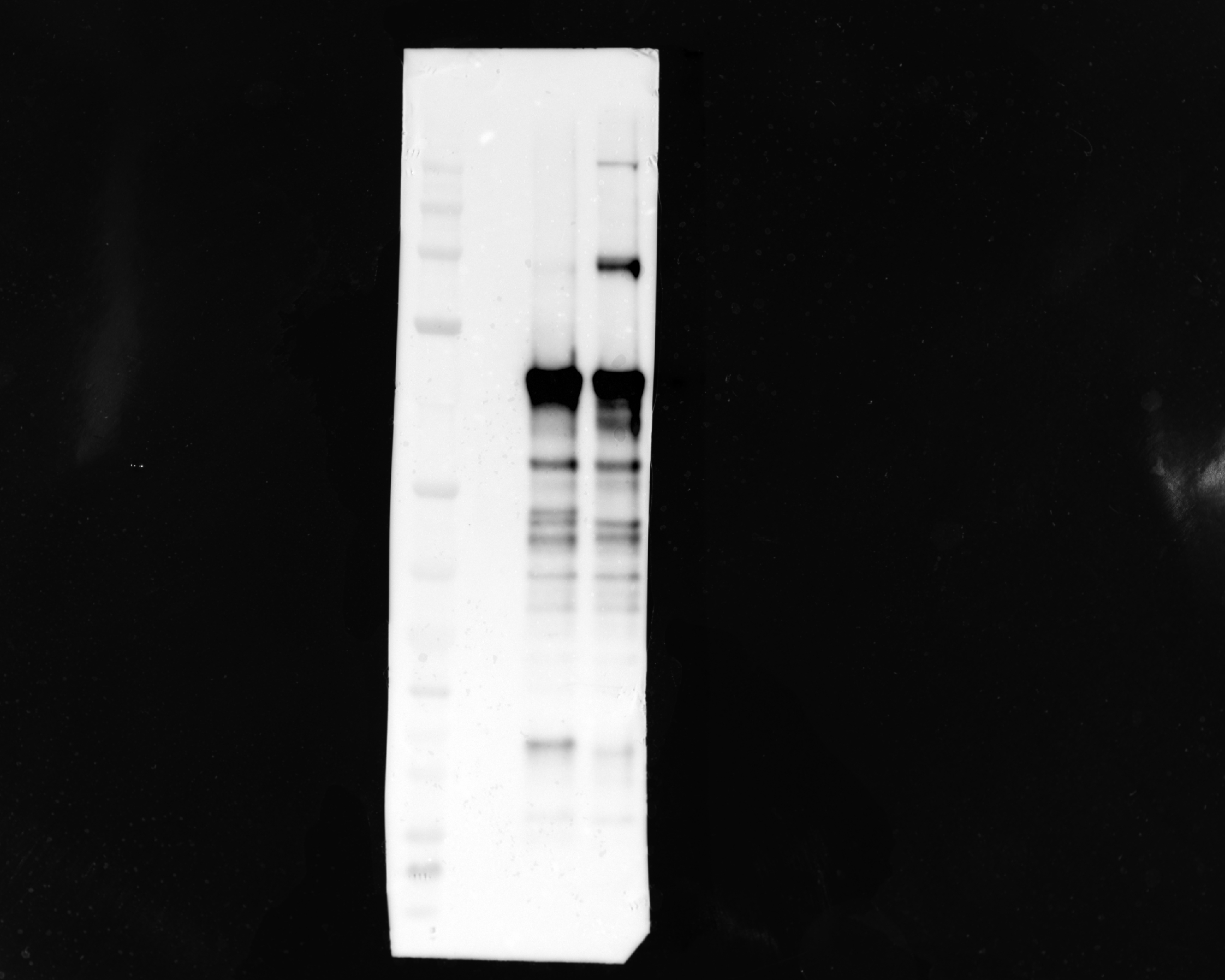


**Suppl 5**

**TEWL**

Measurements of TEWL were performed at room temperature (25±2°C) and 40±10% humidity. The values were recorded when stabilized approximately 10 seconds after the probe had been placed on the skin. TEWL was measured three times for each mouse and the mean values were obtained. All data are presented as the median of 3 repeated recordings.

**Suppl 6**

**Electron microscopy**

For transmission electron microscopy (TEM), specimens were prefixed in 2% glutaraldehyde in 0.1 M phosphate buffer (pH7.4) and then postfixed in 1% OsO_4_ in the same buffer. The dehydrated specimens were embedded in an Epon-Araldite mixture. Ultra-thin sections (approximately 80 nm) were cut (ULTRACAT OmU_4_, REICHERT-JUNG) vertical to the surface and were stained with 2% uranyl acetate for 5 min, and 0.3% lead citrate for 3 min. Transmission electron microscopy (TEM) observations were carried out using a JEM-1400Plus (JEOL) at an acceleration voltage of 80 kV.
